# Editorial: Drug-resistant *Mycobacterium tuberculosis*

**DOI:** 10.3389/fcimb.2023.1215294

**Published:** 2023-05-19

**Authors:** Helena I. M. Boshoff, Digby F. Warner, Ben Gold

**Affiliations:** ^1^ Tuberculosis Research Section, Laboratory of Clinical Immunology and Microbiology, National Institutes of Health, Bethesda, MD, United States; ^2^ Molecular Mycobacteriology Research Unit, Department of Pathology and Institute of Infectious Disease and Molecular Medicine, University of Cape Town, Cape Town, South Africa; ^3^ Wellcome Centre for Infectious Diseases Research in Africa, University of Cape Town, Cape Town, South Africa; ^4^ Department of Microbiology & Immunology, Weill Cornell Medicine, New York, NY, United States

**Keywords:** *Mycobacterium tuberculosis*, tuberculosis, drug resistance, phenotypic resistance, drug tolerance, drug discovery, antimicrobial resistance (AMR), drug synergy

## Introduction

Tuberculosis (TB) is anticipated to regain its position as the leading cause of death globally due to an infectious agent, having transiently ceded that dubious accolade to SARS-CoV-2/COVID-19 (WHO, 2022a). In 2021, 1.6 million deaths were attributed to TB despite the availability of WHO-approved regimens that can cure 85% of patients in 4-6 months (WHO, 2022b). Drug-resistant TB, which is notoriously difficult to treat, accounted for about half a million of the 6.4 million new TB cases in 2021 (WHO, 2022b). Multidrug-resistant TB (MDR-TB), defined as resistance to at least rifampicin and isoniazid, currently requires 6-9 months’ treatment. The duration of antibiotic therapy can increase beyond 12 months for extensively drug-resistant TB (XDR-TB), which is MDR-TB plus resistance to a fluoroquinolone, and bedaquiline or linezolid (WHO, 2022b). Treatment success for MDR-TB is less than 60%, while for XDR-TB it is estimated to be much lower at ~39% (Chakaya et al., 2021). In this Research Topic, 91 authors from locations across the globe contributed 17 reviews and 3 primary research articles covering Drug Resistant *Mycobacterium tuberculosis* (Mtb). Consistent with the complexity of the problem, the collection comprises a diversity of approaches towards understanding, characterizing, and combatting anti-TB drug resistance. With the United Nations General Assembly due to hold its second high-level meeting on the fight against TB in September this year, this collection offers a timely perspective on the threat posed to global health by this major source of antimicrobial resistant (AMR) infections.

## Genetic drivers of persistence and the evolution of resistance

The number of new chemical entities (17) currently approved for clinical trials alone or in combination with selections from 9 existing anti-TB drugs represents a major improvement on previous years (Edwards and Field, 2022; Fernandes et al., 2022). We know, however, that *Mycobacterium tuberculosis* (Mtb), the bacterial cause of TB, has developed resistance to all drugs in the clinic. This sobering realization emphasizes not only the need to develop new drugs and regimens, but also the importance of characterizing the mechanisms that enable the pathogen to acquire drug-resistance or to survive chemotherapy despite apparent genetic susceptibility. Jones et al. review progress in our understanding of how Mtb evolves drug resistance, often through a stage in which the bacillary population is tolerant of chemotherapy or enriched for persister organisms. The authors appraise the genetic underpinnings of drug tolerance, and their associations with the formation of heterogeneous bacterial populations characterized by differences in their states of DNA replication or repair, transcription, translation, metabolism, and compound efflux. Notably, this heterogeneity manifests between patients, during disease progression within a single patient, and even within and between lesions in the same lung. Liebenberg et al. discuss the factors that affect the development of drug-resistant TB, the transmission of resistant disease, the importance of diagnosis of drug resistance, and the implications for public health management. The authors review many of the mechanisms that are associated with the evolution of drug resistance, including the role of toxin-antitoxin systems in generating persistent cells. They also assess aspects of host-mediated physiology that create environments favoring the development of drug-tolerant populations, including the development of mycobacterial biofilms at certain sites of disease. As discussed by Jones et al., the ability to evolve genetic drug resistance is lineage-dependent, further complicating any models used to predict this process. Once drug resistance has developed, the fitness of the resistant cells can be ameliorated by compensatory mutations that impact the function of the drug target or the metabolic pathways perturbed by the mutated target.

The proportion of differentially culturable cells in TB patients undergoing chemotherapy might offer important insight in the presence *in vivo* of persisters in heterogeneous Mtb populations. In their contribution, Peters et al. report that TB patient sputum harbors a significant fraction of bacilli which do not grow on standard solid agar media. These organisms (variously referred to as “differentially culturable”, “differentially detectable”, or “viable but nonculturable”) can, however, be detected by limiting dilution in liquid media with addition of culture filtrates – which may in some cases harbor growth enhancing components that improve the detection of these cryptic populations. They are also observed in patients on chemotherapy even when traditional solid agar colony counts fall below the limit of detection, and they are found in a sizeable proportion of patients upon completion of chemotherapy. Though not a measure of treatment success, measuring differentially culturable bacteria in patients during chemotherapy may provide an indication of the bacterial load. Gordhan et al. report that the number of differentially culturable Mtb may provide a better measure of bacterial numbers in sputum than solid agar, especially in patients with low bacterial numbers at the onset of disease, such as HIV-coinfected patients. Notably, these authors found that resuscitation promoting factors present in culture filtrates did not promote liquid growth of MDR Mtb strains when compared to rifampicin mono-resistant strains, for which higher numbers of bacilli were detected in the absence of growth promoting factors.

## The metabolism of Mtb that drives the evolution of persistent cells and of drug resistant cells

The propensity for mycobacterial populations to contain sub-populations of cells – in some cases even individual/single cells – in different physiological, metabolic and/or replicative states is increasingly recognized as a major contributor to mycobacterial pathogenicity, as well as an impediment to a full understanding of relevant metabolism of the mycobacteria. Put simply, the genomic clonality which typifies many Mtb populations obscures a capacity for phenotypic diversity that historically has been underestimated. Considering the specific case of antibiotic persistence, Shultis et al. pose key questions around the etiology and characteristics of persister mycobacteria (Where do they come from? Are they all created equally)? and the barriers hindering research into these phenomena. In their article, Eoh et al. argue that metabolomics, by providing detailed insights into the molecular compositions of cells, offers key advantages over other techniques through its ability to identify metabolic differences associated with, or causal of, specific phenotypes in genetically identical populations. Their focus on the role of metabolism in drug tolerance and resistance in Mtb is elaborated by Samuels et al., who consider evidence that changes in central carbon metabolism can promote drug tolerance, identifying metabolic pathways as potential drug targets to inhibit the development of drug tolerance and enhance the efficacy of current anti-mycobacterial therapeutics. Taking a broader view which incorporates both clinical and wet lab settings, Mishra and Saito analyze the consequences of heterogeneity in mycobacterial culturability and growth rates on the use and interpretation of mycobacterial culture data. Reinforcing key themes from these papers, Singh et al. consider the specific case of resistance to fluoroquinolones which, given their role as second-line anti-TB agents, threatens the ability to therapeutically limit the progression of MDR-TB to XDR-TB.

The dependence on multidrug combinations as standard therapy for both drug-susceptible and drug-resistant TB highlights the urgent need to expand the existing, approved TB drug arsenal through the identification of new compounds and combinations. In their article, Tomasi and Rubin provide insights into the genetic tools available to improve the identification and prioritization of new drug targets, including those that might potentiate existing drugs. Poulton and Rock similarly consider the potential for chemical-genetics to improve understanding of intrinsic drug resistance in Mtb and how it might be disarmed in developing potent new antitubercular therapies. The importance of looking beyond enzymes and structural proteins as potential new targets is highlighted by Miotto et al., who argue that based on transcriptional profiles following drug exposure, the major transcriptional regulators such as sigma factors and the WhiB, GntR, XRE, Mar and TetR family regulators should be considered as targets for novel interventions.

The advent of inexpensive sequencing technologies has transformed mycobacterial genetics. However, as Nimmo et al. highlight, while genomics has obvious utility in rapidly identifying mutations conferring resistance to new anti-TB drugs, this requires awareness of the fact that the genotype-phenotype correlations which are available for established antibiotics will not be possible where, by definition, new mutations are individually rare. For Stanley et al., though, the real power of genomics extends beyond standard susceptibility determinations to predicting the risk of developing genetic resistance and treatment failure, making precision anti-TB therapies an achievable aspiration through the integration of genetic determinants of antibiotic response into treatment algorithms.

## Approaches to combat persistent and drug-resistant Mtb

Developing antibiotics that tackle drug resistant Mtb requires innovation at many levels. Anti-TB antibiotics have the daunting task of eradicating Mtb that is replicating, phenotypically resistant (often nonreplicating), and/or nonculturable on standard bacteriological agar (differentially culturable/detectable, etc.) (Nathan, 2012; Nathan, 2017). The enormous diversity of host microenvironments and immune chemistries impact Mtb resistance to antibiotics and the development of genetic resistance.

In their review article, Bhagwat et al. explore drug discovery strategies based on evading pre-existing drug resistance and/or development of drug resistance. Some of these strategies include reversing drug resistance with co-treatment of synergizing agents, developing new generations of existing agents that evade resistance, designing drug combinations to thwart resistance mechanisms, polypharmacology, characterizing large numbers of resistant mutants to understand genetic determinants of drug resistance, and implementing early-stage analysis of drug resistance. Roubert et al. provide Pharma’s perspective on strategies to fast-track antibiotic discovery and improve success rates by “upcycling” TB drug discovery. The authors provide blueprints to combat TB drug resistance, including strategies to revisit hit molecules discovered during the golden age of drug discovery, improving drugs currently used to treat TB, modifying TB drugs to evade resistance, and focusing efforts on validated anti-infective targets. In their review article, Greenstein and Aldrich lay out the enormity of the logistical task involved in testing pairwise and multidrug combinations in multiple *in vitro* models thought to mimic host environments and in animal models of TB. Towards addressing this challenge, the authors present an overview of strategies to predict successful drug combinations, including the use of specialized experimental methods such as the hollow fiber model for human PK/PD, as well as the analysis of *in vitro*, animal, and clinical data with computational models and machine learning. Likewise, McNeil et al. highlight the mycobacterial respiratory complexes as a target for drug combinations. Using a careful analysis of data from chemical, genetic, and chemical-genetic experiments, the authors identify points of vulnerability in mycobacterial respiration to exploit with combinations of antibiotics, targets whose genetic or chemical inhibition potentiates the activity of existing drugs, targets whose inhibition is synergistic lethal, and/or combinations anticipated to kill both replicating and nonreplicating Mtb. In their article, Rudraraju et al. propose KasA (beta-ketoacyl synthase) as a high value target to kill both drug-sensitive and drug-resistant TB. In support of their proposal, the authors provide a comprehensive summary of an extensive body of work on KasA genetics, enzymology, inhibitors, structure-based inhibitor design, and KasA inhibitors *in vitro* and in animal models of TB. TB disease occurs through a complex interplay of mycobacterial and host factors, necessitating a broad view of the potential therapeutic approaches that might be adopted. In their primary research article, Glenn et al. test the impact of modulating host inflammation on the formation of differentially culturable Mtb in a murine model of TB. The authors find that the drug dimethyl fumarate, used to manage psoriasis and multiple sclerosis, reduces the numbers of differentially culturable Mtb when dosed alone or when dosed with standard of care TB drugs, rifampicin, isoniazid, and pyrazinamide.

In summary, the primary articles and reviews in this topic collectively emphasize the complexity of drug-resistant Mtb. Translating these concepts to the clinic will require further advancing our knowledge of the biology of Mtb and developing new approaches to TB drug discovery.

## Author contributions

All authors listed have made a substantial, direct, and intellectual contribution to the work and approved it for publication.
